# Molecular Profile and Matched Targeted Therapy for Advanced Breast Cancer Patients

**DOI:** 10.3390/curroncol30020191

**Published:** 2023-02-18

**Authors:** Rosa Falcone, Pasquale Lombardi, Marco Filetti, Alessandra Fabi, Valeria Altamura, Giovanni Scambia, Gennaro Daniele

**Affiliations:** 1 Phase 1 Unit, Fondazione Policlinico Universitario A. Gemelli IRCCS, 00168 Rome, Italy; 2 Department of Experimental Medicine, Sapienza University of Rome, 00185 Rome, Italy; 3 Unit of Precision Medicine in Breast Cancer, Scientific Directorate, Fondazione Policlinico Universitario A. Gemelli IRCCS, 00168 Rome, Italy; 4 Department of Life Science and Public Health, Università Cattolica del Sacro Cuore, 00168 Rome, Italy

**Keywords:** next-generation sequencing, breast cancer, targeted therapy, genomic

## Abstract

(1) Background: Precision oncology is opening new treatment opportunities for patients suffering from solid tumors. In the last two decades, the advent of CDK4/6 inhibitors, immunotherapy, and antibody–drug conjugates (ADC) improved survival outcomes for advanced or metastatic breast cancers (BC). Nevertheless, some patients progress to approved therapies and still maintain good clinical conditions. (2) Methods: With the aim to estimate the accrual rate to experimental precision oncology treatments, we collected molecular and clinical characteristics of BC patients evaluated at Phase 1 Unit of Fondazione Policlinico Gemelli. Clinical data were retrieved from hospital records. Molecular analysis was performed using Next-Generation Sequencing (NGS) FoundationOne CDx on tissue or blood. (3) Results: Among the 38 BC patients referred to our unit, 35 completed the genomic analysis. All patients were female with advanced (mean number of metastatic sites: 3, range 1–6) BC. Median age at our evaluation was 52 (IQR, 48–59). ECOG PS was good in 97% of the study population, although heavily pre-treated (median number of systemic treatments: 5, IQR 3–7). Half of referred patients were HR^+^/HER2^−^ BC, with 39% triple negative breast cancer (TNBC). NGS testing was performed on relapsed disease among most (71%) participants, in particular lymph nodes and soft tissue. Liquid biopsy was requested in 23% of cases. The median time from sample collection to NGS testing was 1 month and from diagnosis 54 months. The median value of mutations, VUS, and TMB were 6, 11, and 5, respectively. *TP53*, *PIK3CA*, *BRCA2*, *ESR1*, and *RAD21* were the genes with the highest number of molecular alterations. In 5 patients (14%), the molecular analysis was helpful to assign targeted therapy in the context of clinical trials with a median progression-free survival of 5 months. (4) Conclusions: HR^+^/HER2^−^ and TNBC were the most frequent subtypes referred for NGS testing. Tissue biopsy of relapsed disease was feasible in 71% of cases. The molecular analysis offered a new treatment opportunity in 14% of patients. The real benefit of these treatments remains to be evaluated in larger cohorts.

## 1. Introduction

Breast cancer (BC) is a complex and heterogeneous disease [1], including several subtypes differing for clinical behavior, prognosis, and response to therapies. The traditional molecular classification of BC (luminal A, luminal B, triple negative, HER2 enriched) has been incorporated into clinical care practice [2]. In contrast, the genomic landscape of advanced disease is an emerging issue investigated by next-generation sequencing (NGS) technologies. Some researchers combined genomic data with clinical details in the effort to identify those molecular alternations that mediate progression or drug resistance and to support targeted therapy decisions [3]. Indeed, tumor sequencing results can guide clinical trial enrolment or identify drug opportunities in individual patients. Detailed analysis of the genome of BC offers the chance to personalize therapy with several limitations: availability of targeted agents, interpretation of genomic alterations and their roles (drivers versus passengers) and agreeing on rules to prioritize actionable findings. Moreover, the spatial and temporal tumor evolution represents an important clinical issue [4]. Archival primary tumor tissue cannot be assumed to be representative of the advanced disease genomic profile and poses a clinical challenge in regard to acquisition of multiple longitudinal tissue biopsies [5]. 

The interpretation of the genomic findings and the indication for personalized therapy remain a matter of debate, with an increasing number of dedicated molecular tumor boards (MTBs) and the attempt to automize the results [6,7]. MTBs take advantage of skills and professionalism of physicians, biologists, and bioinformatics. They are not easy to integrate. In several countries, such as Italy, NGS analysis is heterogeneous in terms of geographical distribution and diagnostic performance, with the urgent need to implement harmonization and laboratory network [8,9]. Moreover, agreement in targeted therapy recommendations among MTBs worldwide is not obvious because MTBs differ in terms of scope, composition, and methods [10]. Moreover, the portfolio of experimental therapies available in each hospital and the ability to perform the analyses in clinically acceptable time are two critical points to address.

Over the past 20 years, new classes of agents (CDK 4/6 inhibitors, antibody drug conjugate, PARP inhibitors, immunotherapy) significantly improved survival outcomes of BC patients [11,12] and offered new treatment opportunities for those progressing to standard therapies. Recently, trastuzumab–deruxtecan (a HER2 antibody–drug conjugate) showed impressive results among HER2^+^ advanced BC [13] with 76% of patients alive without disease progression at 12 months. Two PARP inhibitors, olaparib and talazoparib, have been approved for treatment of germline BRCA carriers with metastatic HER2 negative BC. They showed, compared to chemotherapy, improvement in progression-free survival (PFS) and quality of life but no difference in overall survival (OS) [14]. 

Several platform trials explored the efficacy of targeted therapy in solid tumors, with some basket trials focused on BC. The low accrual rate among the screened patients and the low response rate did not carry out to any drug approval so far [15,16]. A few clinical studies evaluated how NGS testing is used for patients with BC in real-world clinical setting [17,18]. The number of physicians ordering NGS testing increased more than 6-fold over a 5-year period (from 2014 to 2019), with triple negative breast cancer representing the most prevalent subtype undergoing the analysis [18]. With the advent of NGS plasma analysis, tissue and plasma-based analyses were equally ordered in the community setting. 

The aim of this mono-institutional experience was to evaluate NGS tumor profiling among BC patients and the percentage of patients that were assigned to clinical trials with precision therapies, based on the genomic findings.

## 2. Materials and Methods

Genomic and clinical characteristics of breast cancer patients evaluated at Phase 1 Unit of Fondazione Policlinico Gemelli and performing a genomic analysis were collected. 

Breast cancers were classified into four primary molecular subtypes: (1) luminal A or HR^+^/HER2^−^; (2) luminal B or HR^+^/HER2^+^; (3) triple negative or HR^−^/HER2^−^; and (4) HER2-positive or HR^−^/HER2^+^. They were defined HR^+^ if, at immunohistochemistry (ICH), at least 1% of the cells expressed ER and/or PgR; HER2^−^ was used when HER2 expression was 0 or low (1+ or 2+). 

All the molecular analyses were performed using Next-Generation Sequencing (NGS) FoundationOne CDx. Tissue samples (primary diagnosis or relapsed tissue) were preferentially used. Blood was used for the analysis if archival tumor tissue was unavailable, considered too old or a new biopsy was considered unfeasible by the physician. 

Formalin-fixed, paraffin-embedded tumor-containing specimens or blood were sent to the commercial molecular pathology laboratory for NGS in the United States. Details listed in the FoundationOne reports, obtained by this laboratory, were used for the analysis. Extracted DNA from tumor samples was subjected to NGS utilizing the hybrid capture-based FoundationOne^®^ CDx assay (Foundation Medicine Inc., Cambridge, MA, USA), as previously described [19]. NGS was conducted for exons of 324 genes and introns of 36 genes (FoundationOne^®^ CDx), which are frequently altered in various solid tumors. The indicated genomic regions were investigated for base substitutions, insertions, deletions, copy number variants, rearrangements, microsatellite instability, and tumor mutational burden (TMB).

To be included in the analysis, patients had to sign an informed consent. For all the patients, demographics and data about the disease and the treatments were collected from health records of the hospital. All the statistical analyses were performed with SPSS v27.0. Student’s *t*-test, Fisher’s exact test, and Mann–Whitney test for categorical variables were used, as appropriate. *p* value < 0.05 was considered significant.

The study was approved by the institutional ethics committee.

## 3. Results

**Figure 1:**
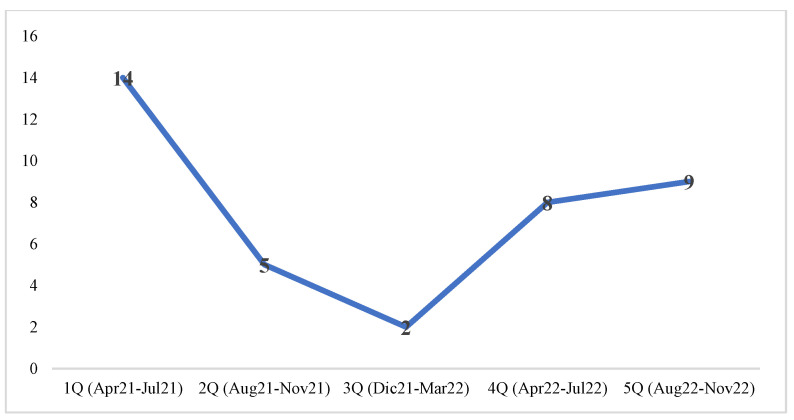
Number of BC patients evaluated for NGS testing over a 20-months period.

From April 2021 to November 2022, we evaluated, for NGS testing, 38 BC patients (Table 1). Three of them did not complete the analysis because of the fast disease progression. The majority of these patients (29, 76%) was internal to our institution and came from Lazio area (31, 79%). The remaining 20% were from the south of Italy. Over a period of 20 months, we saw for NGS testing about 2 BC patients per month, with 38% of patients evaluated in the first quarter (1Q) (Figure 1). A subsequent decline was remarkable during the second and third quarter, remaining on a stable value in the last eight months (4Q and 5Q) of 2022. 

All patients were female, with a median age at diagnosis of 45 (IQR, 42–51) and at our evaluation of 52 (IQR, 48–59). Most patients had HR^+^ cancers (55%) followed by TNBC (39%). ECOG performance status at the time of our evaluation was good (0 or 1) in 97% of the study population although they were heavily pre-treated.

Among the 35 patients who received NGS, the analysis was performed on relapsed disease in 25 patients (71%) and on liquid biopsy in 8 (23%) patients. The diagnostic tissue was used in a minority of patients (n = 2). Plasma-based testing was ordered equally in hormone receptor-positive subtype (5/21, 24%) and TNBC (3/15, 20%).

The median time from sample collection to NGS testing was 1 month and from diagnosis 54 months. The most frequent solid tissues used for NGS testing were breast (7 samples), soft tissue (n = 6), and lymph nodes (n = 6), followed by liver (n = 5), lung (n = 2), and ovary (n = 1). Except for two samples of breast tissue from primary diagnosis, the others were from relapsed disease. 

The median value of mutations, VUS, and TMB were 6, 11, and 5, respectively. Wherever microsatellite status was obtained, there were no cases of instability. *TP53*, *PIK3CA*, *BRCA2*, *ESR1*, and *RAD21* were the genes with the highest number of molecular alterations. The 22 patients with *TP53* mutations carried 25 *TP53* mutations (Table 2). 

Genomic alterations in *TP53* were prevalent among TNBC (50% of all TP53-mutant BC) and observed largely in solid tissue (72% vs. 28% in blood). All patients (100%) with liver metastases had *TP53* mutations. No differences among TP53+ and TP53- BC were identified for other metastatic sites. 

The molecular analysis opened new treatment opportunities for five patients (14%) in the context of clinical trials: two patients (one TNBC, one HR^−^/HER2^+^), because of their high TMB (>16 mut/MB), received immunotherapy; one patient (TNBC) received an AKT inhibitor; two patients (HR^+^/HER2^−^), found to be positive for somatic alterations in *PALB2* and *BRCA2*, received a PARP inhibitor. The median progression free survival was 5 months. For two of these five patients (40%), no other therapies were administered after the experimental drug. 

There were no clinical characteristics significantly different among the group of patients eligible for target therapy (G1, n = 5) and the others (G2, n = 30). Molecular subtypes of BC were homogeneously distributed among the two groups, expect for HER2-enriched subtype that was represented only in G1 (1/5 = 20%). Liquid biopsy and solid tissues were equally used in the two groups (liquid biopsy 20%, solid tissues 80%) for NGS analysis. About the genomic findings, patients in G1 had a higher number of variants of uncertain significance (VUS) compared to G2 (25.25 vs. 11, *p* = 0.0009).

Compared to other advanced solid tumors, among BC patients, the accrual rate for targeted therapies in the context of clinical trials was similar to endometrial cancers (2/20 = 10%), cervical cancers (2/21 = 9.5%), gastro-esophageal cancers (2/12 = 16%), bilio-pancreatic cancers (2/21 = 17%), and lung cancers (4/38 = 10.5%); in contrast, among the ovarian cancers, the accrual rate was very low (1/75 = 1.3%); for colorectal cancers, it was 7%. 

## 4. Discussion

Access to large NGS testing is still a luxury item in the Italian oncologic scenario. Except for academic institutions or research centers, who make advantage of a more or less large home-made NGS panel, many patients do not have the opportunity to have their cancer analyzed from a molecular point of view. Few referral centers, placed in urban metropolitan areas, offer this chance by paying for the analysis or being screened in clinical trials. 

Performing a wider NGS test does not mean receiving targeted therapy. Indeed, we may offer drugs for just a minority of all the researched molecular alterations. Due to the slowness of the bureaucracy, many new drugs, especially in the first phases of the development, are tested in the United States and China, reducing the chances for people from some other countries [20,21]. 

With the aim to estimate the rate of advanced BC patients who really benefit from molecular analysis in terms of new chances of access to targeted therapies, we used a Foundation One NGS test. Over a period of 20 months, we evaluated 38 patients progressing to standard therapies. In five patients (14%), the molecular analysis was helpful to assign targeted therapy to these patients in the context of clinical trials with a median progression free survival of 5 months. With the limit of the small sample size, we found that patients belonging to G1, that are those eligible to target therapies, had a significantly higher number of VUS compared to G2 but, interestingly, they do not differ for number of pathogenic mutations. A VUS is a change in the sequence of a gene which cannot be determined pathogenic (something that causes disease) or benign (something that does not cause disease). The effect of a VUS, whether somatic or germline, remains unclear and its clinical relevance is uncertain. We do not know if there is a correlation between the number of VUS and the value of TMB. Moreover, data about genes carrying a VUS should be collected to provide some correlations. For instance, a recent study showed that, across diverse cancers, VUS in *POL* (DNA polymerase) genes exhibited an additive effect as carriers of multiple VUS and had exponentially increased TMB and prolonged overall survival [22]. 

The identification of an additional marker (TMB), although limited to a single case of a heavy pre-treated HER2-enriched BC, may open new horizons in a disease centered since decades on a unique receptor. 

Most of the evaluated patients (about 75%) were from the surrounding geographic area and followed at our institution. After the initial enthusiasm of medical oncologist to refer BC patients for the genomic analysis (early months of 2021), there was a lack of interest, likely justified by the low accrual rate in clinical trials. After the nadir, over the last eight months of 2022, a stable and intermediate value was reached. With regard to accrual rate, our mono-institutional experience shows similar data to other international trials. Indeed, several platform trials investigated the chance to offer matched targeted therapies based on tissue/blood genotyping. Some of these studies are specific for advanced breast cancers, others included several solid tumors, regardless of the histotype. Low accrual rate was observed both in trials using solid tissue and liquid biopsy. PlasmaMATCH trial assessed the feasibility and clinical utility of circulating tumor DNA analysis to direct therapy in patients with advanced breast cancer, comparing the results with contemporaneous biopsies [16]. Among the 1051 patients who met inclusion criteria and registered, only 136 (13%) entered a cohort and received a pre-planned treatment. *ESR1*, *HER2*, *PTEN*, and *AKT* were the targets for whom matched therapies were proposed. In MEDIOLA trial, a small number of patients (n = 34) with germline *BRCA1* or *BRCA2*-mutated metastatic breast cancer underwent olaparib and durvalumab therapy [23]. In NCI-MATCH, although breast cancers were among the most frequent sequenced tumors, the enrollment rate was very low. Indeed, among the 96 patients evaluated for screening, only 2 (2%) were assigned to therapies [15]. The low rate of accrual supports the idea to encourage the development of new drugs pushing the pre-clinical research in breast cancer field. As long as we have the NGS panel covering more than 300 genes but only 10 drugs to use, the waste of genomic data and economic resources is overwhelming. 

Genomic findings in our analysis are similar to those reported in other experiences [17], with *TP53*, *PIK3CA*, *BRCA2*, *ESR1*, *RAD21*, *CDH1*, *FGFR1*, *ZNF703*, and *FGF3/19* representing the leading alterations. In particular, *TP53* mutations are the most frequent genetic alterations in breast cancer, observed largely in TNBC [24]. Previous studies reported that TP53 mutations are significantly associated with shorter OS and are an independent predictive factor of OS for BC patients [25]. Although TP53 mutations were homogenously distributed throughout the metastatic sites, all patients with liver lesions had TP53 mutations. This finding is not supported by other experiences. Concordance rates for TP53 mutations between breast cancer tissue and plasma are extremely different across studies, which might be dependent on disease subtype, stage, time of sampling, technology used for NGS analysis, and the VAF threshold [26].

Half of referred patients for NGS were HR^+^/HER2^−^ BC, with 39% of TNBC, showing as the need of new treatments for these subtypes is a priority. Although TNBC represents a minority of BC, the large use of NGS in our experience may demonstrate an effort by oncologists to find actionable genes in such a heterogeneous population with poor treatment options and poor outcomes. In contrast, HR^+^/HER2^−^ advanced BC is a common disease with longest overall survival, better outcome, and the chance to underwent multiple treatments over years [27]. In HR^+^/HER2^−^ disease, clinicians and patients may prefer targeted agents over chemotherapy to postpone chemotherapeutics as much as possible. Plasma-based testing was ordered equally in hormone receptor-positive subtype and TNBC: in the first case, the high frequency of bone metastases make it difficult to get diagnostic tissue for genomic analysis. Among TNBC, physicians may favor genomic blood tests, whose results are available in shorter time. 

Our work lacks data about outcomes with target agents outside the clinical trials. Indeed, our institution does not approve off-label requests or compassionate use for cancer patients. A survey among American oncologists showed that even when NGS yielded actionable information, physicians experienced problems obtaining drugs [28]. Younger oncologists, having genomics training and access to a molecular tumor board, are more likely to demand NGS tests [29]. This may partially explain why most of our patients were referred by oncologists working at our institution. 

In conclusion, a deeper understanding of the biological landscape in breast cancer combined with wider access to early- and late-phase clinical studies, equally distributed all over the country, genomic trainings among oncologists with the chance to be part of molecular tumor boards, and a unique and faster national approval for trials may contribute to increase the opportunity of cures for BC patients, regardless of the area or country where they live.

The integration of NGS together with other high-throughput techniques (CNV data, methylation data, miRNA expression data), both at tissue and blood level, will provide stronger evidence to support the choice of a targeted agent rather than another.

## Figures and Tables

**Table 1:** Clinical and molecular characteristics of BC patients.

	N = 38 (%)
Gender female male	38 (100%) -
Ethnicity white other	37 (97%) 1 (3%)
Age at NGS testing *median, IQR*	52, 48–59
Histology HR^+^/HER2^−^ HR^+^/HER2^+^ HR^−^/HER2^+^ TNBC other	20 (53%) 1 (2.6%) 1 (2.6%) 15 (39%) 1 (2.6%)
N metastatic sites mean (min–max)	3 (1–6)
N previous TR median, IQR	5 (3–7)
Concurrent medications median, IQR	3 (1–4)
Charlson comorbidity index (CCI) median, IQR	8 (7–9)
ECOG PS 0 1 ≥2	19 (50%) 18 (47%) 1 (3%)
Tissue for NGS solid tissue blood na	27 (71%) 8 (21%) 3 (8%)
N mutations median, IQR	6 (4–10)
TMB (mut/MB) median, IQR	5.06 (2.5–10.1)
N VUS median, IQR	11 (8–14)
Microsatellite status MSS MSI-H na	21 (55%) - 17 (45%)
Associated mutations: *TP53* *PIK3CA* *BRCA2* *ESR1* *RAD21 * *CDH1* *FGFR1* *ZNF703 * *FGF3/19*	22 (58%) 9 (24%) 8 (21%) 7 (18%) 6 (16%) 5 (13%) 5 (13%) 5 (13%) 5 (13%)

N: number; TR: treatment; PS: performance status; TMB: tumor mutational burden; VUS: variant of uncertain significance; MSS: microsatellite stability; MSI-H: microsatellite instability-high.

**Table 2:** TP53 genomic findings and clinical data.

TP53	Tissue	Histology
R213*	blood, soft tissue	TNBC
Y220C	lymph node	TNBC
LOSS EXONS 2–9	lymph node	HR^+^/HER2^−^
G302fs*41	soft tissue	HR^−^/HER2^+^
S241C	breast	TNBC
R248Q	lymph node	TNBC
V157D	blood	TNBC
P152fs*14	breast	TNBC
F270C	breast	TNBC
E336_L350del	lung	HR^+^/HER2^−^
H193D	blood	HR^+^/HER2^−^
M237I	blood	HR^+^/HER2^−^
P278A	blood	HR^+^/HER2^−^
R273C	liver	TNBC
splice site 919+1G>A	blood	HR^+^/HER2^+^
C176Y	blood	HR^+^/HER2^+^
R342*	breast	TNBC
C176F	liver	HR^+^/HER2^−^
loss	liver	HR^+^/HER2^−^
E326fs*11	liver	HR^+^/HER2^−^
S121fs*25	lymph node	HR^+^/HER2^−^
R273C	soft tissue	HR^+^/HER2^−^
R248W	liver	HR^+^/HER2^−^
R249M	liver	HR^+^/HER2^−^
C275F	soft tissue	TNBC

TNBC: triple negative breast cancer; HR: hormone receptor; HER2: human epidermal growth factor receptor 2.

## Data Availability

Data are available upon request to the corresponding author.

## References

[1] Testa U., Castelli G., Pelosi E. (2020). Breast Cancer: A Molecularly Heterogenous Disease Needing Subtype-Specific Treatments. Med. Sci..

[2] Goldhirsch A., Winer E.P., Coates A.S., Gelber R.D., Piccart-Gebhart M., Thürlimann B., Senn H.-J. (2013). Personalizing the treatment of women with early breast cancer: Highlights of the St Gallen International Expert Consensus on the Primary Therapy of Early Breast Cancer 2013. Ann. Oncol..

[3] Hempel D., Ebner F., Garg A., Trepotec Z., Both A., Stein W., Gaumann A., Güttler L., Janni W., DeGregorio A. (2020). Real world data analysis of next generation sequencing and protein expression in metastatic breast cancer patients. Sci. Rep..

[4] Kimbung S., Loman N., Hedenfalk I. (2015). Clinical and molecular complexity of breast cancer metastases. Semin Cancer Biol..

[5] De Mattos-Arruda L., Sammut S.-J., Ross E.M., Bashford-Rogers R., Greenstein E., Markus H., Morganella S., Teng Y., Maruvka Y., Pereira B. (2019). The Genomic and Immune Landscapes of Lethal Metastatic Breast Cancer. Cell Rep..

[6] Russo A., Incorvaia L., Capoluongo E., Tagliaferri P., Galvano A., Del Re M., Malapelle U., Chiari R., Conte P., Danesi R. (2022). The challenge of the Molecular Tumor Board empowerment in clinical oncology practice: A Position Paper on behalf of the AIOM- SIAPEC/IAP-SIBioC-SIC-SIF-SIGU-SIRM Italian Scientific Societies. Crit. Rev. Oncol. Hematol..

[7] Tamborero D., Dienstmann R., Rachid M.H., Boekel J., Lopez-Fernandez A., Jonsson M., Razzak A., Braña I., De Petris L., Yachnin J. (2022). Author Correction: The Molecular Tumor Board Portal supports clinical decisions and automated reporting for precision oncology. Nat. Cancer.

[8] Marchetti A., Barbareschi M., Barberis M., Buglioni S., Buttitta F., Fassan M., Fontanini G., Marchiò C., Papotti M., Pruneri G. (2021). Real-World Data on NGS Diagnostics: A survey from the Italian Society of Pathology (SIAPeC) NGS Network. Pathologica.

[9] Pinto C., Biffoni M., Popoli P., Marchetti A., Marchetti P., Martini N., Normanno N. (2021). Molecular tests and target therapies in oncology: Recommendations from the Italian workshop. Future Oncol..

[10] Koopman B., Groen H.J., Ligtenberg M.J., Grünberg K., Monkhorst K., de Langen A.J., Boelens M.C., Paats M.S., von der Thüsen J.H., Dinjens W.N. (2021). Multicenter Comparison of Molecular Tumor Boards in The Netherlands: Definition, Composition, Methods, and Targeted Therapy Recommendations. Oncologist.

[11] Jacobs A.T., Castaneda-Cruz D.M., Rose M.M., Connelly L. (2022). Targeted therapy for breast cancer: An overview of drug classes and outcomes. Biochem. Pharmacol..

[12] Keenan T.E., Tolaney S.M. (2020). Role of Immunotherapy in Triple-Negative Breast Cancer. J. Natl. Compr. Canc. Netw..

[13] Cortés J., Kim S.-B., Chung W.-P., Im S.-A., Park Y.H., Hegg R., Kim M.H., Tseng L.-M., Petry V., Chung C.-F. (2022). Trastuzumab Deruxtecan versus Trastuzumab Emtansine for Breast Cancer. N. Engl. J. Med..

[14] Tung N., Garber J.E. (2022). PARP inhibition in breast cancer: Progress made and future hopes. NPJ Breast Cancer.

[15] Flaherty K.T., Gray R., Chen A., Li S., Patton D., Hamilton S.R., Williams P.M., Mitchell E.P., Iafrate A.J., Sklar J. (2020). The Molecular Analysis for Therapy Choice (NCI-MATCH) Trial: Lessons for Genomic Trial Design. J. Natl. Cancer Inst..

[16] Turner N.C., Kingston B., Kilburn L.S., Kernaghan S., Wardley A.M., Macpherson I.R., Baird R.D., Roylance R., Stephens P., Oikonomidou O. (2020). Circulating tumour DNA analysis to direct therapy in advanced breast cancer (plasmaMATCH): A multicentre, multicohort, phase 2a, platform trial. Lancet Oncol..

[17] Bruzas S., Kuemmel S., Harrach H., Breit E., Ataseven B., Traut A., Rüland A., Kostara A., Chiari O., Dittmer-Grabowski C. (2021). Next-Generation Sequencing-Directed Therapy in Patients with Metastatic Breast Cancer in Routine Clinical Practice. Cancers.

[18] Sturgill E.G., Misch A., Lachs R., Jones C.C., Schlauch D., Jones S.F., Shastry M., Yardley D.A., Burris H.A., Spigel D.R. (2021). Next-Generation Sequencing of Patients with Breast Cancer in Community Oncology Clinics. JCO Precis Oncol..

[19] Frampton G.M., Fichtenholtz A., A Otto G., Wang K., Downing S.R., He J., Schnall-Levin M., White J., Sanford E., An P. (2013). Development and validation of a clinical cancer genomic profiling test based on massively parallel DNA sequencing. Nat. Biotechnol..

[20] Marchesi E., Monti M., Nanni O., Bassi L., Piccinni-Leopardi M., Cagnazzo C. (2018). New requirements for phase I trials: A challenge for Italian clinical research. Tumori.

[21] Cagnazzo C., Nanni O., Arizio F., Franchina V., Cenna R., Tabaro G., Vannini F., Procopio G., Gori S., Di Costanzo A. (2020). Phase I studies: A test bench for Italian clinical research. Tumori.

[22] Ying J., Yang L., Yin J.C., Xia G., Xing M., Chen X., Pang J., Wu Y., Bao H., Wu X. (2021). Additive effects of variants of unknown significance in replication repair-associated DNA polymerase genes on mutational burden and prognosis across diverse cancers. J. Immunother. Cancer.

[23] Domchek S.M., Postel-Vinay S., Im S.-A., Park Y.H., Delord J.-P., Italiano A., Alexandre J., You B., Bastian S., Krebs M.G. (2020). Olaparib and durvalumab in patients with germline BRCA-mutated metastatic breast cancer (MEDIOLA): An open-label, multicentre, phase 1/2, basket study. Lancet Oncol..

[24] Bertheau P., Lehmann-Che J., Varna M., Dumay A., Poirot B., Porcher R., Turpin E., Plassa L.-F., de Roquancourt A., Bourstyn E. (2013). p53 in breast cancer subtypes and new insights into response to chemotherapy. Breast.

[25] Andrikopoulou A., Terpos E., Chatzinikolaou S., Apostolidou K., Ntanasis-Stathopoulos I., Gavriatopoulou M., Dimopoulos M.-A., Zagouri F. (2021). TP53 mutations determined by targeted NGS in breast cancer: A case-control study. Oncotarget.

[26] Garrido-Navas M.C., García-Díaz A., Molina-Vallejo M.P., González-Martínez C., Lucena M.A., Cañas-García I., Bayarri C., Delgado J.R., González E., Lorente J.A. (2020). The Polemic Diagnostic Role of *TP53* Mutations in Liquid Biopsies from Breast, Colon and Lung Cancers. Cancers.

[27] Meegdes M. (2023). Real-world time trends in overall survival, treatments and patient characteristics in HR+/HER2− metastatic breast cancer: An observational study of the SONABRE Registry. Lancet Reg. Health Eur..

[28] Smith M.L. (2020). NGS testing use and results: A survey of U.S. oncologists. J. Clin. Oncol..

[29] Freedman A.N., Klabunde C.N., Wiant K., Enewold L., Gray S., Filipski K.K., Keating N.L., Leonard D.G., Lively T., McNeel T.S. (2018). Use of Next-Generation Sequencing Tests to Guide Cancer Treatment: Results from a Nationally Representative Survey of Oncologists in the United States. JCO Precis Oncol..

